# Leigh syndrome: MRI findings in two children

**DOI:** 10.2349/biij.6.1.e6

**Published:** 2010-01-01

**Authors:** AL Kartikasalwah, Ngu LH

**Affiliations:** 1 Department of Diagnostic Imaging, Kuala Lumpur Hospital, Kuala Lumpur, Malaysia; 2 Department of Genetics, Kuala Lumpur Hospital, Kuala Lumpur, Malaysia

**Keywords:** Leigh syndrome, MRI, SURF1, mitochondrial

## Abstract

Leigh syndrome is a progressive neurodegenerative disorder of childhood. The symmetrical necrotic lesions in the basal ganglia and/or brainstem which appear as hyperintense lesions on T2-weighted MRI is characteristic and one of the essential diagnostic criteria. Recognising this MR imaging pattern in a child with neurological problems should prompt the clinician to investigate for Leigh syndrome. We present here two cases of Leigh syndrome due to different biochemical/genetic defects, and discuss the subtle differences in their MR neuroimaging features.

## INTRODUCTION

Leigh syndrome (also termed subacute, necrotising encephalopathy) is a progressive neurodegenerative disorder of childhood with an estimated incidence of 1:40,000 births [1]. Clinically, Leigh syndrome is characterised by psychomotor delay or regression, muscular hypotonia, brainstem signs (especially strabismus, nystagmus and swallowing difficulties), ataxia, pyramidal signs, respiratory insufficiency, lactate acidemia and acute deterioration after common infections. In most cases, dysfunction of the respiratory chain enzymes is responsible for the disease, which could be due to defects in either mitochondrial or nuclear DNA. Despite its considerable clinical, genetic and biochemistry heterogeneity, the basic neuropathological features in children affected by Leigh syndrome are almost identical; which are focal, bilateral, and symmetric necrotic lesions associated with demyelination, vascular proliferation and gliosis in the brainstem, diencephalon, basal ganglia, and cerebellum [2]. MR imaging can demonstrate these brain pathologies and therefore plays an essential role in the diagnosis of Leigh syndrome [3-5].

## CASE REPORTS

### Case 1

**Figure 1:**
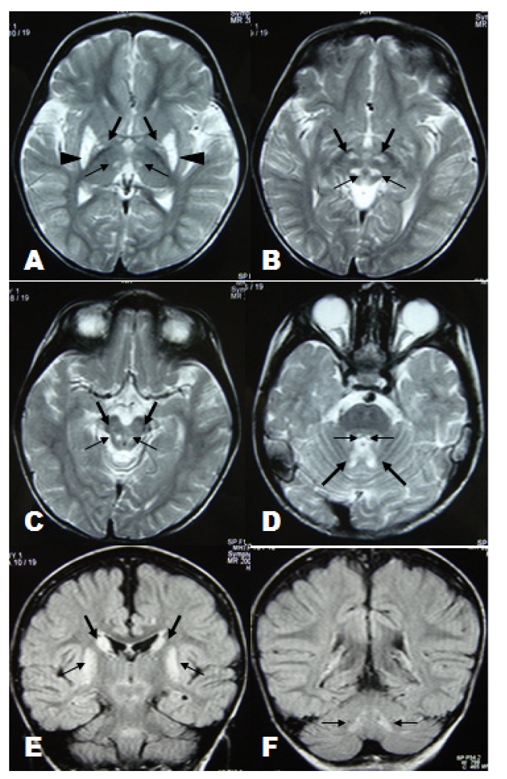
(A): Axial T2-weighted image through the basal ganglia shows symmetrical hyperintense lesions involved thalamic posteromedial ventral nuclei (thin arrow), globus pallidi (thick arrow) and putamina (arrowhead). (B-D): Axial T2-weighted images through the brainstem show symmetrical involvement of reticular formation of midbrain (thin arrow in B), subthalamic nuclei (thick arrow in B), substantia nigra (thick arrow in C), dorsal midbrain (thin arrow in C) central tegmental tracts (thin arrow in D) and cerebellar nuclei region (thick arrow in D). (E-F): Coronal FLAIR images show symmetrical involvement of head of caudate nuclei (thick arrow in E), putamina (thin arrow in E) and dentate nuclei (thin arrow in F).

This boy, the first child of nonconsanguineous parents, had an uneventful perinatal history and normal development until 21 months when he presented with regression. He slowly lost the ability to walk and eventually could no longer walk at 3 years old. At 3 ½ years old, he presented with intractable vomiting and breathing difficulties which required mechanical ventilation. He had persistent metabolic acidosis with markedly raised blood lactate. His clinical findings included truncal ataxia, nystagmus, muscular hypotonia, increased deep tendon reflexes and bilateral Babinski sign. MRI of the brain showed foci of discrete, bilaterally symmetric lesions in the basal ganglia, brain stem and dentate nuclei (Figure 1). Respiratory chain enzymes study in cultured fibroblasts showed severe reduction in Complex IV activity (Table 1). Genetic study found two mutations in SURF1 gene (a heterozygous splice site mutation c.751+1A>G and a heterozygous deletion c.756_757delCA). He was treated with coenzyme Q_10_ and vitamins without much success. He continued to deteriorate and died of respiratory failure at 4 ½ years old during an episode of chest infection. No autopsy was performed.

**Table 1:** Clinical, biochemical and MR imaging findings

	**Case 1**	**Case 2**
**Clinical findings**		
Age of onset (month)	21	19
Psychomotor delay/regression	+	+
Muscular hypotonia	+	+
Pyramidal signs	+	+
Extrapyrimidal signs	+	-
Ataxia	+	-
Respiratory insufficiency	+	-
Nystagmus/opthalmoplegia	+	+
Episodic intractable vomiting	+	-
Swallowing dysfunction	+	-
Deterioration during acute febrile illness	+	+
**Biochemical findings**		
Blood lactate *(normal <2.4mmol/L)*	4.8 - 5.9	5.9
Respiratory chain enzyme activities in cultured fibroblast*		
Complex I *(normal 261-1,051 mU/UCOX)*	589	**212**
Complex II *(normal 536-1,027 mU/UCOX)*	605	681
Complex III *(normal 1,270-2,620 mU/UCOX)*	2,040	1,744
Complex IV *(normal 680-1,190 mU/UCOX)*	**96**	846
Complex V *(normal 217-1,736 mU/UCOX)*	913	980
Citrate synthase *(normal 144-257mU/mg)*	227	207
**Genetic defects**	*SURF1* gene	unknown
**Neuroimaging findings**		
Caudate nuclei	+	-
Putamina	+	-
Globus pallidi	+	-
Thalami	+	-
Subthalamic nuclei	+	-
Substantia nigra	+	+
Reticular formation	+	-
Central tegmental tracts	+	+
Medulla	-	-
Cerebellum	+	-

+, present; -, absent; * Respiratory chain enzyme activity is measured in mU/unit citrate synthase.

### Case 2

**Figure 2:**
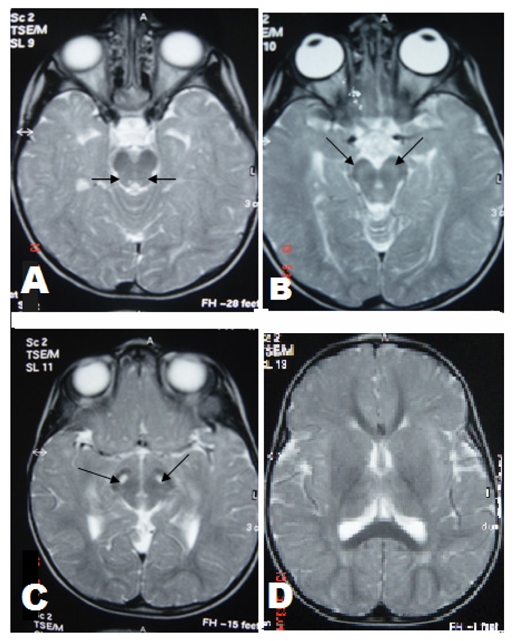
(A-C): Axial T2-weighted images through the brainstem show symmetrical hyperintense lesions involving central tegmental tracts (thin arrow in A), substantia nigra (thin arrow in B and C). (D): Axial T2-weighted image through basal ganglia shows putamen, globus pallidus and thalamus were not involved.

This boy, the 3^rd^ child of nonconsanguineous parents, presented with developmental delay. At 18 months he had yet to be able to walk independently and had no meaningful verbal expressions. Following a febrile illness at 19 months old, he showed regression. There was nystagmus, left eye divergent squint, mild muscular hypotonia and increased deep tendon reflexes on clinical examination. MRI of the brain showed bilaterally symmetric lesions in the basal ganglia and brain stem (Figure 2). The blood lactate was persistently elevated. Respiratory chain enzymes study in cultured fibroblasts showed severe reduction in Complex I activity (Table 1). Mitochondrial DNA analysis did not find any pathogenic mutation. The underlying genetic defect remained to be elucidated. When he was last reviewed at 2½ years, his condition was stable and he was able to walk with broad-based gait.

## DISCUSSION

Leigh syndrome is the most common clinical phenotype of mitochondrial disorders in childhood. The diagnostic criteria are (1) progressive neurological disease with motor and intellectual developmental delay; (2) signs and symptoms of brainstem and/or basal ganglia disease; (3) raised lactate levels in blood and/or cerebrospinal fluid (CSF); and (4) characteristic symmetric necrotic lesions in the basal ganglia and/or brainstem [1].

The most characteristic neuroradiological findings in Leigh syndrome are bilateral, symmetric focal hyperintensities in the basal ganglia, thalamus, substantia nigra, and brainstem nuclei at various levels on T2-weighted MRI. These high T2 signals on MRI reflect the spongiform changes and vacuolation in the affected brain structures [3-5]. Often, the basal ganglia are affected before the brainstem. The upper brainstem followed by lower brainstem would be affected with the progression of the disease. Involvement of lower brainstem indicates advanced stage of the disease and the occurrence of respiratory failure and sudden death. In most patients the cerebral white matter is generally only involved in late stages of the disease. Occasionally, patients may have atypical neuroimaging features such as diffuse supratentorial leukodystrophy, unifocal or multifocal infarctions, diffuse or focal cortical atrophy, or predominant cerebellar atrophy [2,6].

Considering that Leigh syndrome is the consequence of a number of different biochemical and genetic defects that affect many aspects of mitochondrial function, we could expect to find some variability of the MR imaging findings among these patients. In Case 1, the underlying biochemical abnormality was due to a severely decreased Complex IV enzyme activity with the residual enzyme activity only 14% of the lower normal limit. This was due to loss-of-function mutations of *SURF1*, which is a nuclear gene that encodes a protein with putative that is probably involved in Complex IV assembly or stabilisation [7]. In Case 2, the Leigh syndrome was caused by an isolated Complex I deficiency due to a yet to be elucidated genetic defect, most probably a nuclear gene defect. In both cases, there were symmetrical brain lesions involving the brainstem. Case 1 had additional lesions in the basal ganglia, subthalamic nuclei and cerebellum. Our observation on Case 1 is in harmony with a few recent studies that have suggested that symmetric T2 prolongation involving the subthalamic nuclei corresponds with Complex IV deficiency caused by SURF1 mutation [3-5]. It was interesting to note that Case 2 had brainstem involvement prior to basal ganglia.

Our experience suggested that bilateral symmetric T2 prolongation involving multiple brainstem nuclei/structures associated with basal ganglia abnormalities in a child with neurological problems should prompt the clinician to consider Leigh syndrome and conduct further investigations such as measurement of blood and/or CSF lactate, and respiratory chain enzymes activities. Neuroradiological discriminative observation is very useful in guiding the clinicians for the most appropriate enzymatic and genetic study in their patients.
